# Peripheral clinic versus centralized laboratory-based Xpert MTB/RIF performance: Experience gained from a pragmatic, stepped-wedge trial in Botswana

**DOI:** 10.1371/journal.pone.0183237

**Published:** 2017-08-17

**Authors:** Tefera Agizew, Rosanna Boyd, Ndwapi Ndwapi, Andrew Auld, Joyce Basotli, Sambayawo Nyirenda, Zegabriel Tedla, Anikie Mathoma, Unami Mathebula, Chawangwa Lesedi, Sherri Pals, Anand Date, Heather Alexander, Thomas Kuebrich, Alyssa Finlay

**Affiliations:** 1 Center for Disease Control and Prevention, Gaborone, Botswana; 2 Division of Tuberculosis Elimination, Center for Disease Control and Prevention, Atlanta, Georgia, United States of America; 3 Ministerial Strategic Office, Ministry of Health, Gaborone, Botswana; 4 Division of Global HIV and Tuberculosis, Center for Disease Control and Prevention, Atlanta, Georgia, United States of America; Central University of Tamil Nadu, INDIA

## Abstract

**Background:**

In 2011, the Botswana National Tuberculosis Program adopted World Health Organization guidelines and introduced Xpert MTB/RIF (Xpert) assay to support intensified case finding among people living with HIV enrolling in care. An evaluation was designed to assess performance under operational conditions to inform the national Xpert scale-up.

**Methods:**

Xpert was implemented from August 2012 through November 2014 with 13 GeneXpert instruments (GeneXpert) deployed in a phased approach over nine months: nine centralized laboratory and four point-of-care (POC) peripheral clinics. Clinicians and laboratorians were trained on the four-symptom tuberculosis screening algorithm and Xpert testing. We documented our experience with staff training and GeneXpert performance. Test results were extracted from GeneXpert software; unsuccessful tests were analysed in relation to testing sites and trends over time.

**Results:**

During 276 instrument-months of operation a total of 3,630 tests were performed, of which 3,102 (85%) were successful with interpretable results. *Mycobacterium tuberculosis* complex was detected for 447 (14%); of these, 36 (8%) were rifampicin resistant. Of all 3,630 Xpert tests, 528 (15%) were unsuccessful; of these 361 (68%) were classified as “error”, 119 (23%) as “invalid” and 48 (9%) as “no result”. The total number of recorded error codes was 385 and the most common reasons were related to sample processing (211; 55%) followed by power supply (77; 20%) and cartridge/module related (54; 14%). Cumulative incidence of unsuccessful test was similar between POC (17%, 95% CI: 11–25%) and centralized laboratory-based GeneXpert instruments (14%, 95% CI: 11–17%; *p = 0*.*140*).

**Conclusions:**

Xpert introduction was successful in the Botswana setting. The incidence of unsuccessful test was similar by GeneXpert location (POC vs. centralized laboratory). However, unsuccessful test incidence (15%) in our settings was higher than previously reported and was mostly related to improper sample processing. Ensuring adequate training among Xpert testing staff is essential to minimize errors.

## Background

The Xpert MTB/RIF (Xpert) is recommended as the initial diagnostic test among persons with human immunodeficiency virus (HIV) associated presumptive tuberculosis (TB) or multi-drug resistant TB (MDRTB) [1]. Botswana has the second-highest HIV infection prevalence in the world, with one in four adults infected [2], and similar to other high HIV prevalent settings, TB is a leading cause of mortality in this population [3, 4]. The estimated annual incidence of TB was 385 per 100, 000 population, with a TB/HIV co-infection rate of 60% in 2015 [5].

In 2011, the Botswana Ministry of Health (MoH) adopted World Health Organization (WHO) guidelines and incorporated Xpert into the national TB diagnostic algorithm [6].

The Xpert is a real-time fully automated molecular test, developed on the GeneXpert platform (Cepheid, Sunnyvale, CA, USA), that can detect both *Mycobacterium tuberculosis* complex (MTBC) and rifampicin (RIF) resistance within two hours [7, 8]. The GeneXpert instrument (GeneXpert) operates with modules that are the heart of the analytic system, using patented cartridge-based technology [9]. The module currently on the market uses a new software and cartridge version, in combination referred to as G4, that has potential for reducing non-determinant results and error rates than the earlier version, G3 [10].

WHO advocates for the swift and large-scale implementation of Xpert in high-HIV prevalence settings and settings where MDR TB is prevalent because of its improved diagnostic performance over smear microscopy [11]. However the readiness of intended users and health systems, both in peripheral laboratories and in clinics at the point-of-care (POC), is unclear. A Cochrane review in 2014 by Steingart *et al* suggested that not all peripheral-level laboratories may be able to satisfy the operational requirements recommended for Xpert testing, namely an uninterrupted and stable electrical power supply, temperature control, and yearly calibration of the GeneXpert modules[12]. Following early implementation of Xpert in nine high TB burden countries, a wide range of challenges, including infrastructure limitations, training requirements, sub-optimal Xpert test performance, and non-standard result recording have been reported [13].

The usefulness of Xpert for intensified case finding with high sensitivity and specificity has been demonstrated in controlled studies [14, 15]; however, the usefulness of Xpert is dependent on the GeneXpert yielding valid test results. Evaluating the introduction of GeneXpert and its operation in routine HIV care and treatment settings is recommended before nationwide scale-up [16]. Emerging evidence about the GeneXpert in programmatic settings has shown variability of performance in failure rates and accuracy [17–19]. Apart from the GeneXpert and environmental factors such as power supply and temperature, sputum specimen preparation and cartridge version (G3 vs. G4) are among the factors potentially affecting Xpert failure rate and test validity [10, 13].

We documented our experience with staff training, GeneXpert and Xpert test performance during phased rollout of the thirteen GeneXperts serving 22 HIV care and treatment centers in Botswana, and aimed to address the following operational research questions: (1) what were the challenges experienced during GeneXpert installation and training, (2) among successful Xpert tests, what was the prevalence of MTBC and MTBC with RIF resistance, (3) what was the incidence of unsuccessful Xpert test by reason for unsuccessful test (i.e., “error”, “invalid”, or “no result”), (4) did unsuccessful test incidence differ between and POC-based and centralized laboratory-based GeneXpert, (5) among unsuccessful Xpert tests, what was the percentage due to error and type of error, (6) did incidence of unsuccessful test and error type differ between G3 and G4 cartridges, (7) was there notable trend over time in incidence of unsuccessful test by reason for unsuccessful test, (8) was there an association between unsuccessful test incidence and training level, and (9) what were the challenges maintaining GeneXpert and the effect of delayed calibration on unsuccessful test incidence?

## Methods

**Fig 1:**
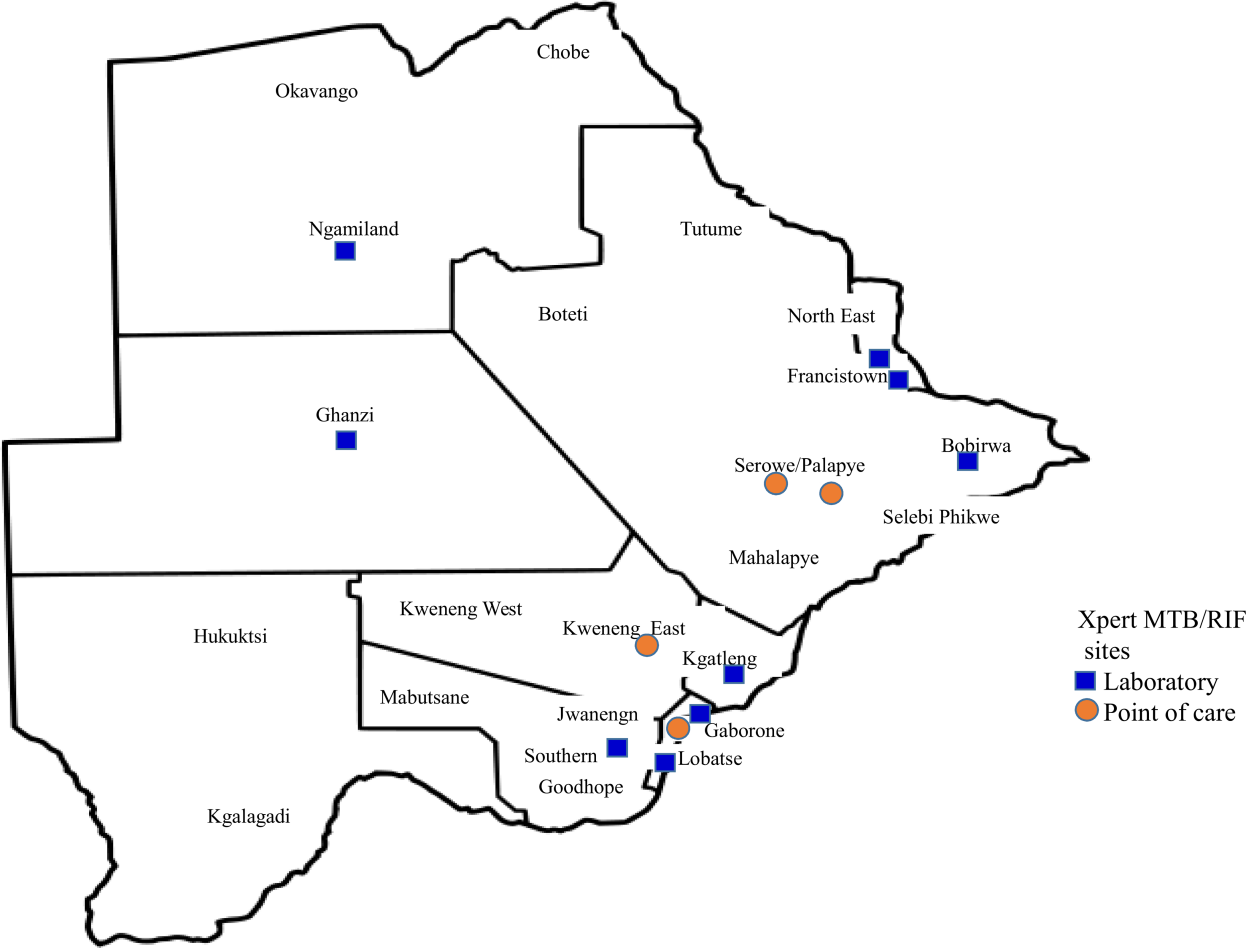
Thirteen GeneXpert instruments sites in Botswana.

Twelve of the 28 health districts with high HIV and TB burden were prioritized by MoH for initial Xpert implementation in routine settings following Botswana National TB guideline revision in 2011 to include Xpert in the TB diagnostic algorithm [6]. Xpert was introduced in a phased manner and prospectively evaluated and monitored, together with Patient enrolment and follow-up, from August 2012 through November 2014. Installation of thirteen GeneXperts occurred over nine months between October 2012 and June 2013 at nine centralized laboratory sites and four POC clinical care sites without on-site laboratory services (shown in Fig 1). One to two GeneXperts were installed per month during this period, each with four modules for a total of 52 modules. The order of GeneXpert placement was randomized. GeneXpert operators included laboratorians at centralized laboratory sites and nurses at POC testing sites.

With introduction of Xpert at implementing districts, two trainings were organised. Training for clinicians consisted of a one-day training curriculum. This curriculum covered standard TB symptom screening, use of the Xpert-based TB diagnostic algorithm [6, 20], techniques to collect good quality sputum specimens, sputum transportation, information about the Xpert test, expected turnaround times, interpretation of test results, and a refresher on the management of TB and MDR TB.

Training for GeneXpert operators consisted of a three-day curriculum and standard operating procedure (SOP) manual. Three to four laboratorians at each centralized laboratory site and three to four nurses at POC sites were trained as operators. The training covered the theoretical basis of the Xpert test, how to operate the instrument, interpretation of results, troubleshooting, and GeneXpert maintenance (daily, weekly, and monthly). The third day of the training was hands-on operational training. All trainees had to pass a competency test before testing patient specimens from study sites.

The GeneXpert was initially installed by a local GeneXpert vendor who provided calibration and maintenance services during the study, Xpert cartridge sales, and cartridge delivery services. Air conditioning units were installed with each GeneXpert to ensure control of room temperature. Uninterruptible power supply (UPS) systems were installed with each GeneXpert to ensure sufficient electrical power to allow completion of in-process sample analysis during power grid outages (up to 30 minutes at sites with back-up generators and up to 2 hours at sites without generators). Xpert cartridges were procured through the same vendor at $18 per cartridge which included the cost of the cartridge, central warehousing, and delivery to sites when requested.

Xpert implementing sites were visited by a laboratory supervisor or senior study staff member quarterly and clinical sites were supervised monthly by study nurse supervisors to support implementation. For quality assurance purposes the laboratory supervisor visited the GeneXpert sites quarterly to assess if SOPs were being followed and to conduct in-service training as needed.

### Data collection and analysis

Data were collected by study staff between August 2012 and November 2014. Xpert test result files (.gxx file format) were archived monthly by GeneXpert operators, extracted from GeneXpert software, and collected quarterly when laboratory supervisors from the referral laboratory conducted supervisory visits. GeneXpert software version 4.0 was used to access test results at a central location and results were compiled quarterly and stored in a Microsoft Excel spreadsheet. Tests conducted for quality assurance or training purposes were excluded from the analysis. Unsuccessful tests were defined as any result of “error”, “invalid” or “no result”. All remaining tests were considered successful which included results as MTBC not detected, MTBC detected plus RIF resistance detected/not detected/intermediate. The frequencies of successful and unsuccessful tests were analyzed and described, including the type of unsuccessful test by site, by Xpert cartridge version (G3 vs. G4), and by quarter of operation.

#### Data analysis

Descriptive statistics were computed in Microsoft Excel and in STATA 14.0 [21]. Robust standard errors were estimated and used in 95% confidence intervals (CIs) for percentages and means. We tested for differences between groups (e.g. POC vs. centralized laboratory tests) using the STATA GLLAMM [22] procedure to fit logistic regression models with clinic as a random effect. Interaction variables were included in these models to test for differential effects over time between POC and centralized laboratory tests.

The study protocol was approved by the Botswana Health Research and Development Committee (May 16, 2012), the US Centers for Disease Control and prevention Institutional Review Board (IRB) (July 19, 2012), and the University of Pennsylvania IRB (June 24, 2012). Patients at study sites were enrolled in the study following the IRB-approved, written, informed consent process. The trial was registered to clinicaltrials.gov (NCT02538952) retrospectively based on the realization of the importance of trial registration.

## Results

### Procedures implemented and challenges experienced during GeneXpert installation and training

A total of 254 clinical personnel at study sites were trained at study initiation to implement the Xpert-based TB diagnostic algorithms using the day-long training curriculum. Of those trained, 77 were medical officers, 126 were registered nurses, 28 were laboratorians, and 23 were staff of other cadres.

The initial three-day trainings for GeneXpert operators at each study site were conducted simultaneously with GeneXpert installation. Forty-six GeneXpert operators were trained, among whom 17 (37%) were nurses at POC sites. During the supervisory visits, an additional 32 GeneXpert operators, including 12 nurses, were trained per request from the implementing sites, resulting in a total of 78 who received the three-day standard MoH-recommended training. During supervisory visits we found that 21 staff members (20 laboratorians and one nurse) who did not receive the standard three-day training were conducting Xpert testing. The reason provided was that qualified staff (i.e., those who received the three-day Xpert training and passed the proficiency test) were not available for Xpert testing due to rotation of laboratorians or nurses. During these instances, where qualified laboratorians or nurses were not available for Xpert testing, laboratories allowed one of the laboratorians or nurses to do the Xpert testing after receiving hands-on half-day training, referred to hereafter as in-house training, without a need to formally pass the competency test.

During our supervisory visits we observed and documented that: (1) every three months laboratorians were rotating from one laboratory section to another and every two years from one laboratory to another, and this led to Xpert testing sites with qualified staff constraints; (2) at two sites, out of the laboratorians conducting Xpert, more than half received only in-house training; (3) operators did not always adhere to SOPs, with frequently observed mistakes including: variable sample and reagent mixing time, inconsistent daily, weekly and monthly GeneXpert maintenance, and inconsistent daily room temperature monitoring. Reminders and demonstrations by supervising study staff were necessary to improve adherence to SOPs; (4) GeneXpert operation at the four POC sites was interrupted more than once because a nurse was not available for testing. Reasons encountered included: (a) heavy nursing workloads and short staffing in busy clinics making it difficult for the nurses to break away to perform the test; (b) inadequate coverage during night shift rotations and leave (night off the following day) schedules; and (c) reluctance to operate the GeneXpert because it was initially perceived as a non-nursing duty.

### Xpert test results

A total of 3630 Xpert tests were performed over 276 instrument-months of operation. Of these, 3102 (85%) were successful. Among all tests run, 447 (12%) resulted in a positive test for MTBC (Table 1), ranging from 1–23% depending on the testing site. Among all 3102 successful tests, MTBC was positive in 14%. Among samples that were positive for MTBC, the proportion with RIF resistance was 8% (36) and 3% (15) were indeterminate.

**Table 1:** Xpert MTB/RIF implementation of results at laboratory and POC in Botswana, October 2012 to November 2014.

Xpert site	Tested N (%)	MTBC‡ Detected		Rifampicin Resistance						Unsuccessful test		Type of unsuccessful test						Valid test	MTBC w/o unsuccessful test
		N	%	Detected		Indeterminate		Not Detected		n	%	Error		Invalid		No result			
				n	%	n	%	n	%			n	%	n	%	n	%	n	%
ATH **	**197 (5)**	**26**	**13%**	**3**	**12%**	**2**	**8%**	**21**	**92%**	**29**	**15%**	**25**	**86%** ** **	**2**	**7%**	**2**	**7%**	**168**	**15%**
AWC **	**641 (18)**	**78**	**12%**	**10**	**13%**	**0**	**0%**	**68**	**87%**	**115**	**18%**	**91**	**79%** ** **	**17**	**15%**	**7**	**6%**	**526**	**15%**
BK8 **	**560 (15)**	**67**	**12%**	**7**	**10%**	**2**	**3%**	**58**	**87%**	**72**	**13%**	**34**	**47%** ** **	**33**	**46%**	**5**	**7%**	**488**	**14%**
BOB **	**200 (6)**	**20**	**10%**	**2**	**10%**	**2**	**10%**	**16**	**80%**	**16**	**8%**	**13**	**81%** ** **	**3**	**19%**	**0**	**0%**	**184**	**11%**
DRM **	**150 (4)**	**11**	**7%**	**1**	**9%**	**2**	**18%**	**8**	**73%**	**12**	**8%**	**9**	**75%** ** **	**3**	**25%**	**0**	**0%**	**138**	**8%**
EXT*	**161 (4)**	**2**	**1%**	**0**	**0%**	**0**	**0%**	**2**	**100%**	**14**	**9%**	**9**	**64%** ** **	**4**	**29%**	**1**	**7%**	**147**	**1%**
GAN **	**166 (5)**	**35**	**21%**	**2**	**6%**	**2**	**6%**	**31**	**89%**	**17**	**10%**	**12**	**71%** ** **	**4**	**24%**	**1**	**6%**	**149**	**23%**
KAD *	**259 (7)**	**32**	**12%**	**2**	**6%**	**2**	**6%**	**28**	**88%**	**38**	**15%**	**16**	**42%** ** **	**22**	**58%**	**0**	**0%**	**221**	**14%**
LMH **	**292 (8)**	**21**	**7%**	**2**	**10%**	**0**	**0%**	**19**	**90%**	**45**	**15%**	**36**	**80%** ** **	**1**	**2%**	**8**	**18%**	**247**	**9%**
MCC*	**397 (11)**	**67**	**17%**	**5**	**7%**	**0**	**0%**	**62**	**93%**	**79**	**20%**	**47**	**59%** ** **	**14**	**18%**	**18**	**23%**	**318**	**21%**
NKO *	**265 (7)**	**28**	**11%**	**1**	**4%**	**0**	**0%**	**27**	**96%**	**50**	**19%**	**44**	**88%** ** **	**3**	**6%**	**3**	**6%**	**215**	**13%**
NRH **	**121 (3)**	**28**	**23%**	**1**	**4%**	**0**	**0%**	**27**	**93%**	**19**	**16%**	**16**	**84%** ** **	**0**	**0%**	**3**	**16%**	**102**	**27%**
SDA **	**221 (6)**	**32**	**14%**	**0**	**0%**	**3**	**9%**	**29**	**91%**	**22**	**10%**	**9**	**41%** ** **	**13**	**59%**	**0**	**0%**	**199**	**16%**
**All sites**	**3630 (100%)**	**447**	**12%**	**36**	**8.1%**	**15**	**3.4%**	**396**	**89.0%**	**528**	**14.5%**	**361**	**68.4%** ** **	**119**	**22.5%**	**48**	**9.1%**	**3102**	**14.4%**

* Point-of-care (POC)

** Centralized laboratory

**‡ MTBC =** *Mycobacterium tuberculosis* complex

Athlon Hospital = ATH, Area W Clinic = AWC, Block 8 Clinic = BK8, Bobonong Primary Hospital = BOB, Deborah Retief Memorial Hospital = DRM, Ext 3 Clinic = EXT, Gantsi Hospital = GAN, Kadimo Clinic = KAD, Letsholathebe II Memorial Hospital = LMH, MCC Clinic = MCC, Nkoyaphiri Clinic = NKO, Nyangabgwe Referral Hospital = NRH and SDA Hospital = SDA

### Incidence and type of unsuccessful Xpert tests

A total of 528 (15%, 95% CI, 12–17%) Xpert test results were recorded as unsuccessful and the cumulative unsuccessful test incidence varied from 8–20% across sites (Table 1). Among all tests conducted, the majority of unsuccessful tests were errors, 361/3630 (10%, 95% CI, 7–13%). Invalid results made up 119/3630 (3%, 95% CI, 2–5%) of all tests conducted and no result were obtained in 48/3630 (1%, 95% CI, 1–3%). The error, invalid, and no result incidence percentages varied by site, ranging from 4–17%, 0–8% and 0–5%, respectively (Table 1). Cumulative incidence of errors was higher than the manufacturer predicted error rate of 5% in 12 out of the 13 sites [23].

### Incidence of unsuccessful Xpert test at POC and centralized laboratory sites

When results were stratified by type of site, unsuccessful test incidence was similar, POC (181/1082, 17%, (95% CI, 11–25%) vs. centralized laboratory (347/2548, 14%, 95% CI, 11–17%), (*p = 0*.*140*).

Error, invalid, and no result cumulative incidence percentages were similar between POC and centralized laboratory sites (Table 2).

**Table 2:** Type of unsuccessful tests by Xpert testing sites.

	POC* N = 1082 (100%)			Centralized laboratory N = 2548 (100%)			
Type of unsuccessful test	n	%	95% CI	n	%	95% CI	*p value*
Error	116	11%	5–21%	245	10%	6–14%	0.870
Invalid	43	4%	1–13%	76	3%	2–6%	0.078
No result	22	2%	0–13%	26	1%	1–2%	0.310

* POC = Point-of-Care

### Incidence of error by type

Table 3 displays the error codes by category based on the most common underlying causes of unsuccessful tests: sample processing, power supply, cartridge/module related, temperature-related, other GeneXpert related [9]. Overall, 385 total errors codes were recorded from 361 error results; for 24 tests more than one type of error code was recorded. The most common type of errors were related to sample processing (codes 2008, 5006 and 5007) (55%), followed by power supply (code 2127) (20%), cartridge/module-related (code 2032 and 5011) (14%), temperature-related (codes 1001, 1002, and 2014) (5%) and other GeneXpert errors (code 2034, 2035 and 1006) (6%). The type of error did not differ significantly between POC and centralized laboratory sites (Table 3).

**Table 3:** Type of Xpert error by GeneXpert site in Botswana, October 2012 to November 2014.

Error types by site		Total n = 385*	100%	POC‡ n = 123	100%		Laboratory n = 262	100%	OR§ 95% CI	*p* value
1	Sample processing	211	55%	52	42%		159	61%	1.81 (0.91, 3.58)	0.089
	2008, 5006 and 5007 n (%)									
2	Power supply	77	20%	38	31%		39	15%	0.53 (0.08, 3.64)	0.520
	2127 n (%)									
3	Cartridge/module	54	14%	16	13%		38	15%	0.33 (0.01, 9.62)	0.521
	2032 and 5011 n (%)									
4	Temperature	20	5%	17	14%		3	1%	0.69 (0.18, 2.64)	0.586
	1001, 1002 and 2014 n (%)									
5	Others	23	6%	0	0.0%		23	9%	N/A**	
	1006, 2034 and 2035 n (%)									

* Error codes were 385 since more than one error code was recorded from an error test result

**Insufficient number of error to allow significance testing

§ OR = Odds Ratio

‡ POC = Point-of-Care

Note for Table 3. Xpert unsuccessful tests were defined according to the manufacturer’s specifications, as follows: 1001 = High temp in module, heater component failure, broken fan, dust on filter near fan; 1002 = the actual temp has drifted too far away from set-point; 2008 *=* pressure exceeds maximum pressure acceptable, or failure of Xpert module, mostly caused by sample viscosity; 2014 = The heater/module’s/optical block thermistor failed; 2032 = Ultrasonic horn current could not be tuned properly; 2034 = optical signal from detector n/LED n did not reach expected value; 2035 = ultrasonic failure occurred with n% duty cycle; 5006/5007/2008 = failure of probe check control; mainly associated with sputum viscosity and/or volume, incorrect filling of reaction tube or detection of probe integrity problem; 5011 = loss of tube pressure; invalid = results that occur if the user fails to comply with the advice provided; no result *=* insufficient data were collected because the test was stopped voluntarily or due to electrical failure. Cepheid Jun 2012 [9].

### Comparing incidence of error between G3 and G4 cartridges

The error proportion was similar for tests using G3 and G4 cartridges, (41/432, 9% vs. 320/3198, 10%, *p = 0*.*855)*. However, the proportion of errors of type 5011 (loss of tube pressure) & 2032 (ultrasonic horn could not be tuned properly) was significantly higher with G3 (14/41, 34%) than with G4 (40/344, 12%), *p = 0*.*002*.

### Trend over time in incidence and type of unsuccessful test

**Fig 2:**
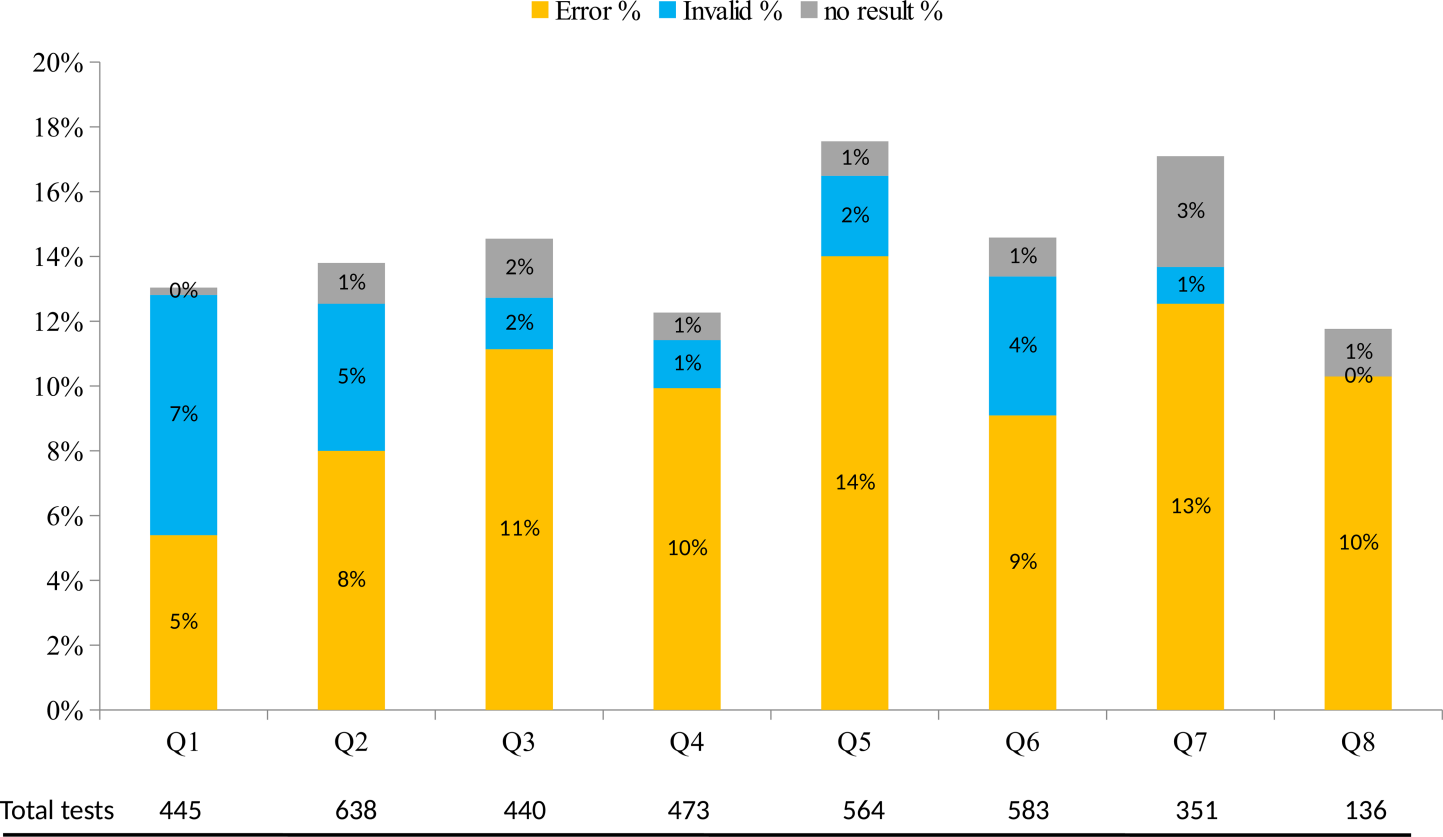
Error, invalid, and no result rates by quarter following initial implementation October 2012 to November 2014. Error rates showed increasing trend over 8 quarters, *p = 0*.*008*. Invalid rates showed decreasing trend over 8 quarters, *p<0*.*001*. No result showed increasing trend over 8 quarters, *p = 0*.*099*. Note for Fig 2. By the time of analysis all 13 sites have experienced at least four quarters. Q5 –eleven sites (ATH, WC, BOB, BK8, DRM, EXT, GAN, KAD, LMH, NRH and SDA). Q6 –ten sites (ATH, AWC, BOB, BK8, DRM, GAN, KAD, LMH, NRH and SDA). Q7. Five sites (ATH, AWC, BK8, KAD and LMH). Q8 –two sites (ATH and KAD).

The overall cumulative unsuccessful test incidence varied by quarter of operation but these differences were not significant *(p = 0*.*570*). As shown in Fig 2, there was a linear decrease *(p<0*.*001)* for the percentage of invalid test and an increase for error tests *(p = 0*.*008)* across the eight quarters. The percentage of tests with “no result” increased slightly but the difference was not significant *(p = 0*.*099)*. There was no significant difference between the incidence of unsuccessful test over time for POC sites compared to centralized laboratory sites *(interaction p = 0*.*338)*.

### Evaluating association between training level and incidence of unsuccessful test or incidence of “error”

Twenty-one percent (21/99) of GeneXpert operators were trained in-house. We examined the effect of in-house operator training on incidence of unsuccessful tests and error, categorizing percentages as 0–10, >10–20, >20–30, >30–40, >40–50 and > 50. The omnibus (overall) tests were not significant for either unsuccessful tests *(p = 0*.*061)* or for error *(p = 0*.*241)*, so we did not do pairwise comparisons between training levels.

### GeneXpert maintenance and the potential effect of delayed calibration on incidence of unsuccessful test

During the course of the study, Xpert testing was interrupted for a number of reasons including: laptop computer stolen, computer not functioning, trained staff not available to operate the test, and power supply/electricity outages. Multiple UPS devices failed over the study period. During 276 GeneXpert instrument-months of operation, 6/13 (46%) UPS devices failed and required replacement. All failed UPS devices were located at centralized laboratory sites except one, at KAD clinic that is a POC site. Error code 2127 (n = 77) resulting from power interruptions during a test run occurred among 9/13 (69%) Xpert testing sites. At KAD despite reported UPS failure no power-related error was reported. Fifty (50/77, 65%) test errors with code 2127 occurred at sites where the UPS devices had no record of failure.

From October 2012 through November 2014, annual calibrations were done for 15 GeneXperts, each with four modules (13 GeneXperts received their first annual calibration, and 2 out of the 13 GeneXperts received their second annual calibration). Among these 5/15 (33%) were calibrated on-time and 5/15 (33%) within one month of the calibration due date. We defined a calibration as “delayed” if calibration was done beyond one month of the due date. Delayed calibrations occurred for 5/15 (33%), with delays ranging from 1–11 months. Within the delayed calibration period, a total of 175 (5%) Xpert tests were conducted yielding 32/175 (18%) unsuccessful test incidence, 95% CI 7–42%) vs. 510/3502 (15% unsuccessful test incidence, 95% CI 12–17%) recorded using GeneXperts with no delay of calibration *(p = 0*.*134)*. Error incidence was 14% (24/175), 95% CI 3–45% vs. 10% (337/3455), 95% CI 7–13%, among samples tested with GeneXpert calibration that was delayed and not delayed, respectively, *(p<0*.*050)*. Error codes recorded with tests conducted with delayed calibration were: Five 2008 (sample processing related), six 2032 (cartridge/module related) seven 2127 (power supply related), one 5006 and five 5007 (both sample processing related).

Calibrations were contracted out to the sole local Cepheid vendor who was unable to deliver on-time service because of human resource constraints and inadequate maintenance tracking. Six modules failed over the course of the study and all were replaced with refurbished modules.

## Discussions

Before countrywide scale-up, we implemented Xpert and assessed its performance prospectively under programmatic conditions in service of HIV care and treatment centres in Botswana. While integrating Xpert into routine systems required considerable investment in human resource training and infrastructure, and was associated with several challenges, several of which were difficult to anticipate. Xpert implementation in Botswana was feasible in both POC and laboratory settings. Obstacles included failed UPS systems, rapid and regular rotation of personnel leading to the need for “in-house” training curricula, and delayed calibration of GeneXperts by local vendors. Lessons learned during this pilot implementation of Xpert continue to inform nation-wide scale-up with the expectation that implementation challenges will decline as the health system adjusts to the new diagnostic assay. However, continued monitoring is necessary to inform the long term use of Xpert in Botswana.

In this study the cumulative incidence of unsuccessful Xpert tests on sputum from HIV-infected presumptive TB patients was 15%. Recent publications, involving multiple countries with high TB burden in Africa, Asia, and Europe, demonstrated overall Xpert unsuccessful test incidence of 5–10% [12, 17, 24, 25]. Two of these studies reported Xpert test results from laboratory settings. The other two included POC facilities in their report, one programmatic and the other in a controlled trial setting. Overall, unsuccessful test incidence in our study was one and half-fold higher than incidence reported from programmatic settings and three-fold higher than incidence reported in a multicentre clinical trial from Southern Africa [13, 24].

Similar to Creswell *et al* and Ardizzoni *et al* [13, 25], about two-thirds of the unsuccessful tests in our study were recorded as error, followed by invalid (22%) and no result (9%). More than half of the errors were related to sample preparation processing or wrong sample volume added to the cartridge, suggesting that most of the unsuccessful tests were related to GeneXpert operators, rather than the instrument. In the present study there was a non-significant higher error rate when in-house trained operators were testing specimens possibly suggesting operator-related error might partly explain high incidence of unsuccessful test. We are in agreement with Raizada *et al* that such errors could be minimized if trained Xpert operators follow SOPs correctly, handle sample preparation appropriately, and improve transfer of required minimum sample volumes into the Xpert cartridge. Regular monitoring visits from Xpert trainers is critical to prevent potential errors [17].

On the other hand, despite back-up generators and UPS, one in five errors were linked to power supply suggesting that alternative sources of reliable power supply should be explored. The use of solar panels has been successfully piloted in India and Uganda and might work well in Botswana [17, 26]. One in seven errors was cartridge or module-related. Creswell *et al* reported 11–44% incidence of errors due to loss of signal or tube pressure (5011 code), and these results were from data collected before version G4 was widely available (March 2013) in the market [13]. Recent publications, however, reported that cartridge-related errors, particularly code 5011 due to signal loss error, were considerably reduced following introduction of the G4 cartridge [10, 25]. Consistent with the recent studies, error incidence was 34% with G3 but 12% with G4 in our study. This improvement is reassuring but further reductions in error rates are still needed. There were relatively few temperature-related errors (5%) in spite of summer temperatures regularly reaching >35°C in Botswana. Sites were well-prepared in advance by equipping the Xpert locations with air conditioning; however improved monitoring and documentation of testing room temperature and GeneXpert maintenance (daily, weekly, and monthly) would be helpful to ensure optimal operating conditions.

There are only a few studies that have compared POC and centralized laboratory-placed GeneXpert performance. Theron *et al* in the Southern African trial reported a similar unsuccessful test incidence at a POC and centralized laboratory (5% vs. 6%, *p = 0*.*22*) [21]. Similarly, the present study did not find any difference between POC and centralized laboratory. In the present study we were able to document further the type of unsuccessful test by testing site; the overall proportion of test with an error, invalid or no result was similar in both settings.

The 17% unsuccessful test incidence at POC in our setting, in contrast, was higher than that reported from Cambodia, which reported 12%. In Cambodia, unlike our study, the POC GeneXpert was operated by laboratorians [13].

Furthermore, in our study, power supply and temperature related issues (i.e., environmental factors) were lower at the centralized laboratory sites while sample processing issues (i.e., operator-related factors) were higher at the centralized laboratory sites. None of the differences were statistically significant (Table 3). To clarify factors affecting GeneXpert performance at POC vs. centralized laboratory, further studies focusing on potential determinants of GeneXpert performance are warranted.

Over time, within eight quarters following introduction of Xpert testing, the error rate increased and the rate of invalid result rate decreased. The incidence of unsuccessful test over time was not affected by testing sites. It is possible that the continuous training offered to study clinicians on quality specimen collection techniques improved their ability to collect quality specimens and minimized invalid test results, which are often related to poor sample quality (e.g., food particles or blood in the sample). The inadequacy of maintaining trained Xpert operators, expressed by the need for more training, may explain increasing error rates over time. Creswell *et al* and Ardizzoni *et al* emphasized the need for retraining of GeneXpert operators due to staff turnover [13, 25]. Likewise, during the two-year study period the initially trained GeneXpert operators in our study were largely not retained, mostly due to transfer to other sections of the laboratory or to other facilities. We observed that more than half of GeneXpert operators had to be trained during follow-up supervision or trained in-house. Operators need for retraining or additional training included troubleshooting and error interpretation, suggesting performance and operator skill could be improved. A one-time (initial) Xpert training is unlikely to be sufficient. Refresher training and annual competency assessments could be incorporated into supervision visits. New staff should receive adequate training and it should be planned in anticipation of regular staff turnover.

About five percent of the total specimens were tested using GeneXpert with delayed calibration. While there was no significant difference in the overall unsuccessful test incidence, the actual error incidence was higher when specimen was tested using GeneXpert with delayed calibrationFurther evaluation of the effect of delayed GeneXpert calibration on Xpert performance could provide useful information on the importance of on-time calibration.

Our study has some limitations. First, although we controlled for clustering by specifying random effect for study facilities, we did not control for other site- and operator-specific factors. For example, there may have been an association between unsuccessful test and certain GeneXpert operator. We were not able to assess the type and frequency of individual test results by GeneXpert operators because operators often shared the same password so there was no reliable identifying record available. Second, information about interruptions of Xpert testing while processing, down time when the GeneXpert was not functioning, and reasons for interruption of testing and for how long interrupted were not systematically recorded. Third, room temperature at Xpert testing sites was not consistently recorded. Although a log sheet was placed at each site to capture this information and GeneXpert operators were taught how to fill out the form, information was rarely recorded sufficiently to allow quantification of downtime and causes. Fourth, six of our modules that had not passed calibration were replaced by refurbished modules. South Africa has reported concerns about the performance of refurbished modules being associated with a higher incidence of unsuccessful tests [27]. However, we did not track which specimens were tested by refurbished modules and therefore are unable to evaluate if refurbished modules had higher incidence of unsuccessful tests than other modules. Last but not least, where qualified laboratorians or nurses were not available for Xpert testing due to staff turnover in-house trained Xpert operators, without a need to formally pass the competency test, conducted some tests that might have negative effect on Xpert test results.

In conclusion, Xpert was successfully introduced in the Botswana programmatic setting, with all sites fully set up per required timelines and site infrastructure. During Xpert implementation challenges associated with introducing a new POC diagnostic test into the health system were observed, particularly nurses not always available to perform Xpert testing at POC or showed occasional reluctance, as operating the GeneXpert was perceived as a non-nursing duty, until the task-sharing role was received well with further consultation. The other critical factors needing attention were on-time maintenance and operation support of the new equipment at all testing sites. The observed unsuccessful test incidence was high compared to other published reports. Repeating tests from leftover specimens has potential to provide valid test results, with correct adherence to SOPs, especially for sample processing errors. Ensuring adequately trained staff are retained and scheduling regular refresher trainings including competency assessments at testing sites will be important to minimize errors.

Our study presented GeneXpert performance, described error linked with codes to help elucidate causes, stratified the result by type of testing site, and examined trends over time. Identified successes and challenges in our setting were important when Botswana MoH was considering countrywide scale up of Xpert. Over 116 countries in the world have already invested in Xpert implementation by December 2014 [28] and these findings contribute to initial reports of GeneXpert operation in resource-limited settings.
